# Women’s empowerment as self-compassion?: Empirical observations from  India

**DOI:** 10.1371/journal.pone.0232526

**Published:** 2020-05-13

**Authors:** Tannistha Samanta

**Affiliations:** Humanities & Social Sciences, Indian Institute of Technology, Gandhinagar, Gujarat, India; Institute of Economic Growth, INDIA

## Abstract

Although ICPD brought about an international consensus on the centrality of women’s empowerment and gender equity as desired national goals, the conceptualization and measurement of empowerment in demography and economics have been largely understood in a relational and in a family welfare context where women’s altruistic behaviour within the household is tied either to developmental or child health outcomes. The goals of this study were twofold: (1) to offer an empirical examination of the household level empowerment measure through the theoretical construct of self-compassion and investigate its association with antenatal health, and (2) to ensure robust psychometric quality for this new measure. Drawing data from the nationally representative, multi-topic dataset of 42, 152 households, *India Human Development Survey*, IHDS II (2011–2012), the study performed a confirmatory factor analysis followed by an OLS estimation to investigate the association between a self-compassionate based empowerment and antenatal care. Empowerment was shown to be positively and significantly associated with antenatal care with significant age and education gradient. A woman’s married status, her relation to the household head and joint family residence created conditions of restricted freedom in terms of her mobility, decision making and sociality. The empowerment measure showed inconsistent associations with social group affiliations and household wealth. The study provided an intellectual starting point to rethink the traditional formulations of empowerment by foregrounding its empirical measure within the relatively unexplored area of social psychology. In the process it addressed measurement gaps in the empowerment-health debate in India and beyond.

## 1. Introduction

While the International Conference on Population & Development (ICPD) [1] brought about an international consensus on the centrality of (women’s) empowerment and gender equity as desired national goals, the conceptualization and measurement of empowerment in demography and economics have been largely understood in a relational and in a family welfare context where women’s altruistic behaviour (or domestic duty) within the household (e.g. decisions on cooking, household ration, etc.) is tied either to developmental or child health outcomes. As such, the women’s empowerment scholarship is replete with studies that demonstrate pathways through which women achieve certain virtuous development goals related to microcredit programs, poverty alleviation and sustainable development [2–4]. Notwithstanding the policy relevance of these studies, the focus remains on enhancing instrumental gains over individual self-efficacy or as Cornwall [5] puts it incisively, “empowerment is treated as a destination reached through development’s equivalent of motorways: programmes rolled out over any terrain” (p.342) with no or limited attention to women’s individual realities. By shifting the empirical gaze to woman’s self-efficacy related constructs, the current paper builds on and contributes to the scarce body of scholarship that goes beyond the empowerment-development paradigm.

Given this intellectual and policy background, this study asked if empowerment can be understood in relation to one’s own health and wellbeing. In particular, this study proposed to offer an alternative definition of the household level empowerment measure by including dimensions of self-compassion: a non-judgmental attitude towards one’s own self that includes feelings of awareness, recognition and assertiveness; since research has demonstrated that self-compassion has strong positive association with personal empowerment which in turn generates health gains.

While women’s empowerment is conceptually understood as a “process” [6] and interrelated terms such as agency, status and autonomy have been variously used in the literature, the lack of adequate data across time makes the measurement of quantifying gains in women’s power and agency problematic. Additionally, scholars note that even if longitudinal data on women’s level of agency/status were available, “the behavioral and normative frontiers that define appropriate indicators for measuring empowerment are constantly evolving” [7]. In this light, Kabeer [6] suggests that “preconditions” of empowerment which include economic, social and human resources, can create the potential for certain demographic outcomes (or “achievements”) including health behaviors and outcomes. Thus, in Kabeer’s sense, the exercise of power or agency in the presence of resources is the “process” of empowerment making it possible to measure women’s empowerment with cross-sectional data [8]. Subsequently, this paper relied on the *India Human Development Survey* (2011–2012) to examine how women’s reproductive (captured through antenatal care) health contributes to empowerment at personal and household levels.

Premised on Basu & Koolwal’s [9] unconventional treatment of women’s empowerment that distinguishes “altruistic (instrumental) versus selfish (self-interest) notions of female autonomy” (p.19), this study developed an empowerment measure rooted in social-psychology (self-compassion) to investigate the health-empowerment debate (discussed in the next sections). The focus on (own) health is crucial as Basu & Koolwal [9] argue that “when women’s autonomy is put to the service of meeting their own health and other needs, it is quite possible that there is much resistance” (19) and hence to what extent women can take strategic decisions about their own bodies and health may offer a more “durable” [10] way of understanding women’s expansion of capabilities. The word “resistance” is pertinent here especially in understanding a woman’s ability to pay attention to *herself* in a context where her needs and aspirations remain subservient to patriarchal loyalties of family and community. For example, demographic scholarship from India has shown that women’s access to education and employment (typical routes through which self-efficacy may be realized) can in fact increase her risk of marital violence arising from a patriarchal anxiety of controlling women’s economic independence and mobility [11,12]. Admittedly, Basu and Koolwal’s plea to focus on woman’s *own* health assumes substantive significance in the health-empowerment debate. In particular, Basu & Koolwal [9] utilized the National Family Health Survey-2, 1998–99 [13] data to estimate the effects of household as well as individual characteristics on health outcomes (related to the woman herself and her child/children). The authors’ conceptualization of individual level explanatory factors includes what they call the “self-indulgent” empowerment (variables that capture women’s ability to do things for herself and hence expand her everyday freedoms) as well as those that reflect her capacity to make the best decisions for her family (empowerment as responsibility). Subsequently, these explanatory variables enter their logistic regression models as binary controls where they demonstrate the strongest statistical associations with the (self-indulgent) empowerment variables and women’s own health. It is this finding as well as the creative articulation of the “self” (indulgent or altruistic) that largely motivates the current paper, but it differs in its empirical treatment (described in later sections). Also, the current paper combines pathways of body related constructs, economic freedoms and community participation in its theoretical model, an aspect that has received limited attention in prior studies (including in Basu & Koolwal’s piece). As such, the current paper’s contribution lies in expanding the theoretical development of a survey-based empowerment measure that is uniquely focused on self that simultaneously encompasses robust psychometric qualities.

### 1.1 Theory and background literature

Theoretically, this study drew from two sets of works: one by [14] in-depth qualitative work on women’s empowerment in rural Bangladesh and second, social-psychological constructs on self-compassion [15,16]. In Hashemi & Schuler’s ([14]: cited in [8]) extensive qualitative study on rural Bangladesh, “sense of self” was identified as one of the dimensions of empowerment based on activities that women identified as important in their everyday lives. The authors noted that “increased *assertiveness*-a sign of transformation in a woman’s self-perception” (p. 4, italics added) as an important dimension capturing a “sense of self and vision of a future”. Later, Schuler and colleagues [17] in an effort to reassess the empowerment indicators developed in their earlier [14] study, confirmed the salience of self-efficacy as a reliable way to demonstrate women’s articulacy and confidence in negotiating her everyday life. In another line of inquiry, self-compassion has been shown to have promising potential in relation to personal empowerment [16]; in these sets of studies self-compassion has been used mostly as a determinant for positive and desirable psychological outcomes including emotional resistance, self-esteem, successful aging, coping and positive outcomes following HIV diagnosis. Drawing from a rich body of social psychological research, the authors note that “as a non-judgemental self-attitude” self-compassion unambiguously predicts greater self-improvement and later, they go on to show in their own study that it increases empowerment through personal control, assertiveness, resource access and an overall awareness of discrimination. Neff [15,18] who also developed and validated a scale to measure self-compassion, emphasized on the importance of understanding, awareness and recognition of the self in driving positive health outcomes. Although, the psychological link between the self and demographic/health outcomes cannot be empirically ascertained with the current data, the conceptual framing of self-compassion allows one, as Basu & Koolwal [9] would argue, “to separate out the self-interest value of empowerment from its instrumental (or altruistic) properties” (p. 19). Further, since women are typically known to be more self-critical about themselves than men (suggesting that women may have lower levels of self-compassion, see [19], cited in [15]) understanding empowerment through self-compassion as a theoretical route through which strategic decisions about self are made holds promise for development interventions that aim at making the gains prevail. Taken together, it is evident that a focus on the *self*, is a common and a consistent element from both the theoretical strands. Enjoying leisure and access to media (and by that extension knowledge and overall exposure) would have been ideal candidates to enhance the “sense of self”, but current survey data does not allow for a quantitative analysis based on these dimensions. Ultimately, to be “empowered” in this conceptual model would be to be more aware of their rights and duties to *themselves* and their own welfare-physical, social and political. Finally, important to note that empowerment in this model is understood as a static, individual form and not as a group process. In that sense, the word empowerment here is similar to what [20] terms as autonomy which reflects “the extent to which women exert control over their own lives within families in which they live at a given point in time” (p. 205). While acknowledging the conceptual distinction between empowerment and autonomy (for details see Agarwala & Lynch [21]), for the purpose of this paper, the word empowerment (a more encompassing term) is adopted since ultimately both the terms are used in the empirical literature to operationalize the route to gender equality in the developing world.

### 1.2 Existing gaps and study objectives

While empirical associations between women’s empowerment and health outcomes were established in the demographic scholarship, the concern about how we *cannot* measure women’s autonomy [22] dominated the field for a while. Indirect proxies that were mostly single observable characteristics (e.g. women’s education or labor force participation) were soon found to be highly imperfect, especially when used to examine the antecedents and effects of empowerment (Balk [23] cited in Agarwala & Lynch [20]). Henceforth, scholars have turned to direct proxies which are a combination of observable items characterizing the multidimensional nature of women’s empowerment. While significant progress has been made on both theoretical conceptualization and measurement aspects of women’s empowerment, recent review of literature shows that majority of studies still pay inadequate attention to measurement errors which have implications on the overall empowerment construct. For example, [24] note that one of the fundamental strengths of CFA approaches to construct validation is that the resulting estimates are adjusted for measurement error (unlike in other models where factor models are specified under the assumption that measurement error is random). Again, in a recent systematic review piece by Pratley [25] of 67 articles (retained from a total of 14,584 studies) shows that majority of studies still use summative index as opposed to a method that utilizes theoretical factors to determine its fit with the overall data. This empirical conundrum of utilizing theoretical constructs in a statistically efficient way to measure empowerment is particularly acute in research on developing countries. This can be attributed to data limitations in these countries and in part to a sustained intellectual reluctance to move away from the conventional theorizing and statistical application of this measure. For example, although theoretical persuasions of Kabeer [6], Williams [8], Basu & Koolwal [9] were noteworthy, their statistical operation of the empowerment measure was limited. As indicated earlier, in Basu & Koolwal’s study, although effort was made to tap into the unconventional notions of empowerment by focusing on responsibility and self-indulgence (captured through women’s responses to food consumption patterns, leisure and anemia during pregnancy), the statistical operation of the measure ignored the differential weighting of each of the factors. Meanwhile, the studies of Williams [8], Agarwala & Lynch [20] or Mishra [26] addressed statistical issues related to differential weighting of factors and measurement errors, but the conceptual framing of empowerment remained restricted to typical routes of women’s access to economic resources and household decisions.

The current study has attempted to address these concerns by focusing on two interrelated objectives: *First*, offering a conceptual intervention by examining empowerment through the lens of self-compassion, and *second*, developing this measure while paying attention to statistical robustness and psychometric quality.

## 2. Methods

### 2.1 Data and sample

Data for this study came from the *India Human Development Survey-II* (IHDS II), 2011–2012 which has received IRB approval. Data is publicly available for download at: https://www.icpsr.umich.edu/icpsrweb/DSDR/studies/22626. IHDS is a cross-sectional, nationally representative, multi-topic survey of 42, 152 households (204, 569 individuals) in 1503 villages and 971 urban neighborhoods across 33 states and union territories of India. This survey was led by the researchers at the department of Sociology, University of Maryland College Park, MD and funded substantially by the U.S National Institute of Child and Human Development (For more information about the survey, see: https://ihds.umd.edu/sites/default/files/BriefII.pdf. Household and individual interviews covered topics including health, education, employment, economic status, marriage, fertility and gender relations. The survey followed face-to-face interviews with the household members including the head of the household, an ever-married woman aged 15–49 and youth. Interviews with the ever-married woman (eligible woman) covered questions regarding health, education, family planning, marriage and gender relations in the household and community. On average, eligible women in this sample were 36 years of age (Standard Deviation, s.d.: 9.86), with around 5 years of education (s.d: 4.92) with a mean age of marriage being around 18 years (s.d. 3.72). The eligible women’s file (N = 39,253) in IHDS-II (2011–2012) provided empirical indicators for the theoretical dimensions of empowerment as self-compassion. Taken together, these variables emphasize assertiveness and a focus on self to improve and expand her own capacities.

### 2.2 CFA, self-compassion based empowerment and psychometrics

This study utilized a confirmatory factor analysis (CFA, henceforth) approach to examine the measurement of self-compassionate empowerment. CFA is useful since it “places *a priori* structure on the data and allows the explicit testing of competing hypotheses regarding the measurement properties of indicators thought to reflect the theoretical construct” [20]. Additionally, CFA allows for refining the empowerment measure based on the modification indices. Modification indices indicate how the model might be adjusted to improve its fit. The empowerment measure in this paper was developed based on a systematic review of empirical scholarship with a focus on measurement issues [8,20,25,27]. Initially, the theoretical model included a total of 18 items thought to capture empowerment that privileges self-compassion. Indicators for self-compassionate empowerment that directly or indirectly focus on the self were identified under four dimensions (1) bodily integrity (3 items); (2) economic (3 items); (3) decision making (6 items) and (4) interaction with non-family groups and public visibility (6 items). The identification process was guided by theoretical scholarship as well as gaps in empirical studies. For example, under the dimension of bodily integrity, indicators such as self-reported health, contraceptive practice and fertility choice have been explored. The attempt has been to identify indicators that demonstrate her perception and ability to do things for herself. For example, the empirical scholarship on self-compassion and its unique relationship to the body has been examined in sports studies [28] where women reported an active interest in understanding and taking ownership of their own bodies. Hence, negotiations around fertility and contraceptive choices have been used as body-related constructs that enhance women’s capacities. Again, women’s tolerance of domestic violence is an important indicator to assess her own self-esteem and bodily integrity. However, in the current data, the violence questions were worded in the context of the overall community (*in your community*, *is it usual for husbands to beat their wives in the following situations*) and hence could not be used. Similarly, under the dimensions of economic and decision making, variables were identified that indirectly reflect the woman’s value of self-worth within the household. For example, through questions on “who has the most say” on economic choices (e.g. buying an expensive item, land/property, expenditure on social functions) or her economic freedom (a marker of self-worth) achieved through the routes of financial acknowledgement (e.g. name on ownership papers; bank account). Again, under the dimension of “interaction with non-family groups”, the attempt has been to identify indicators that go beyond domestic duties and demands of kinship ties and represent some degree of physical freedom that the woman might enjoy. It is conceivable that variables such as her participation in the local self-help group, village/ward level administrative meetings or visiting friends on her own represent an enhanced capacity to negotiate the household environment while paying attention to her own social needs, self-worth and wellbeing.

Significantly, literature on self-compassion emphasizes on the theoretical as well as empirical relevance of autonomy, social interactions/support and subjective wellbeing. Hope and colleagues [29] in their study on University freshmen in Canada found a positive relationship between self-compassion and autonomy which in turn affected their goal pursuits and overall subjective wellbeing. Similarly, Toplu-Demirtas et al. [30] in their research on lesbian, gay and bisexual (LGB) in Turkey show how self-compassion mediate s relationship between social interaction and well-being. Although, it has not been empirically established and perhaps not immediately obvious how women’s physical mobility and her participation in local governance bodies are directly associated with self-compassion, it is reasonable to assume that a self-compassionate woman is more aware of her personal environment (through extra-familial social connections/ties and freedom of movement) to make empowering choices for herself; in fact in Neff’s original formulation [15], social connectedness and autonomy were important dimensions of self-compassion. Having said that, it must be noted that the variables chosen for this study are not completely unambiguous in their conceptual rendering of woman’s self-compassion and hence may not readily translate into their wellbeing. This is primarily because most of the scholarship on self-compassion is linked to a range of psychological health indices which are unavailable in the current dataset and perhaps theoretically unattainable in a non-phenomenological research design. Nonetheless, this conceptual ambiguity has been partially addressed by utilizing CFA to examine the associations between the observable items chosen for the overall empowerment measure.

Table 1 presents the list of all items in the original theoretical model under the above four dimensions.

**Table 1:** Original set indicators and dimensions of empowerment as self-compassion in the theoretical model (before CFA).

**Bodily Integrity**
1. In general, would you say your own health is very good, good, okay, poor, very poor
2. Are you and your husband currently using any methods to delay or prevent pregnancy
3. Who has most say in how many children you have
**Economic**
4. Do you have cash in hand to spend on household expenditure
5. Is your name on the ownership or rental papers for your home
6. Is your name there on any bank account
**Decision Making**
7. Who has most say on whether to buy an expensive item such as TV or fridge
8. Who has most say on whether to buy land or property
9. Who has most say on how much money to spend on a social function such as marriage
10. Who has most say on what to do when you fall sick
11. Who has most say in decisions about your work
12. Who chose your husband
**Interaction with non-family groups and public visibility**
13. Can you alone (whether you need permission or not) to the local health center
14. Can you go alone (whether you need permission or not) to go to the home of relatives & friends
15. Are you a member of a Mahila Mandal
16. Are you a member of a self-help group
17. Are you a member of a credit/savings group
18. Have you attended a public meeting/gram sabha called by the village panchayat/nagarpalika/ward committee in the last year

**Source:** India Human Development Survey (IHDS-2, 2011–12)

The original empowerment measure (a composite index) included 18 variables covering the above four dimensions; a confirmatory factor analysis (CFA) was performed and it was found that 11 variables have higher factor loadings than the rest and hence were retained. The final empowerment measure (Cronbach’s alpha = 0.67; mean = 2.61; s.d = 1.82) was an index that took the value from 0 to 11; 0 = indicating either “no” or “no say” in any of the identified variables and a value of 11 = indicating “yes” or a “most say” in all questions. To demonstrate that CFA offers a more “parsimonious understanding of covariation among a set of indicators” [24], an alpha value for all the original 18 items was calculated. It resulted in a score of alpha = 0.52 (as opposed to an alpha = 0.67 with the 11 items following the CFA). Later, to ensure that the CFA-based measure offers an improvement over summative empowerment index, an OLS model estimating the effects of predictors on empowerment (summative index of all the 18 items) was carried out (results not reported here). Results show that a dampening effect on the positive association between empowerment and antenatal care (i.e. unstandardized beta coefficients, b = 0.016* as opposed to b = 0.129*** (CFA adjusted measure).

Additionally, construct validity of this measure was assessed by looking at the distribution of empowerment values (0–11) across the Indian states. The goal was to examine if this measure is consistent with the scholarship on the association between women’s agency and demographic behavior. The differential ranking of the states in terms of gender inequality and subsequently a woman’s agency (or social position) has been shown to be governed by sociological factors including marriage practices (endogamy/exogamy), cultural tolerance of violence, sexual autonomy, freedom of movement and finally, social institutions (e.g. legal/inheritance, lineage and descent systems) that either constrict or facilitate women’s decisions about her own education, paid work, health and family (See [11,31,32]). For example, as observed in previous scholarship the empowerment values in the current study were higher in the north eastern (such as Sikkim (3.26), Manipur 4.24 or Meghalaya 4.68) and the southern states (Kerala 3.77 or Tamil Nadu 3.21) of India when compared to the northwestern states-of Punjab (2.33) or Haryana (2.24) or Uttar Pradesh (2.19) or Gujarat (2.57)-that have values lower than the average value of empowerment of the country as a whole (2.61). Significantly, these state-level differences can be largely attributed to the practice of patriarchy with the northwestern and a few eastern states (e.g. Jharkhand, Bihar) known to be more deeply entrenched in a culture of patriarchal hierarchies of gender and generation resulting in substantially lower levels of education, employment and mobility among women.

Table 2 summarizes the post-CFA retained indicators of empowerment and Table 3 presents the factor loadings in indicators of empowerment. Factors were determined on the basis of loadings generated by the CFA model. Typically, factors with higher loadings (those close to 1 and with higher Eigen values) were selected since it is generally agreed that they fit the data better [8]. To evaluate the model fit, chi2 (χ 2) and comparative fit index (CFI) were used as proposed by [33]. The p-value associated with χ 2>0.05 and the CFI value >0.95 suggesting that the model meets the cut-off for good fit.

**Table 2:** Indicators of empowerment.

**Indicators of Empowerment**	**Mean**	**S.D**
Who has most say about how many children you have	0.253	0.434
Who has most say about what to do when you fall sick	0.233	0.423
Who has most say whether to buy an expensive item	0.108	0.311
Who has most say about whether to buy land or property	0.082	0.274
Who has most say about how much to spend on a social function	0.153	0.36
Can you go alone (or need permission) to the local health center	0.707	0.454
Can you go alone (or need permission) to home of relatives/friends in the village/neighbourhood	0.767	0.422
Whether you are a member of a Mahila Mandal	0.055	0.228
Whether you are a member of a self-help group	0.135	0.342
Whether you are a member of a credit/savings group	0.071	0.255
Whether you have attended any public meeting/gram sabha/ward committee in the last year	0.085	0.279
**Observations**	36,427	

Source: IHDS (2011–12)

**Table 3:** Factor loadings (generated by CFA) for indicators of empowerment.

Factor loading (pattern matrix) for indicators			
**Indicator variables**	**Factor 1**	**Factor 2**	**Factor 3**
Who has most say about how many children you have	0.4721	-0.0751	-0.0213
Who has most say about what to do when you fall sick	0.5403	-0.048	-0.0176
Who has most say whether to buy an expensive item	0.7125	-0.0943	-0.1467
Who has most say about whether to buy land or property	0.75	-0.0992	-0.1599
Who has most say about how much to spend on a social function	0.6793	-0.0723	-0.0824
Can you go alone (or need permission) to the local health center	0.2757	0.1083	0.6224
Can you go alone (or need permission) to home of relatives/friends in the village/neighbourhood	0.2523	0.0623	0.6297
Whether you are a member of a Mahila Mandal	0.1252	0.4798	-0.0603
Whether you are a member of a self-help group	0.0997	0.4542	-0.0757
Whether you are a member of a credit/savings group	0.0772	0.5065	-0.1067
Whether you have attended any public meeting/gram sabha/ward committee in the last year	0.1258	0.4909	-0.0569
**Method**	Principal Factor		
**Observations**	36427		

Source: IHDS (2011–12).

### 2.3 Variable descriptions and covariates

#### 2.3.1 Outcome variable

The outcome variable for this study was empowerment as self-compassion (the description of this had been provided in section 2.2).

#### 2.3.2 Independent variable

The independent variable for this study was antenatal care, which is a reasonable proxy of whether the woman is attentive of her own health needs during her reproductive journey which in turn has the ability to improve her self-efficacy. While associations between women’s empowerment and fertility behavior (e.g. contraception) have been fairly extensive (see [34] for a recent review), the relationship between women’s empowerment and pregnancy related healthcare has received inadequate attention. Further, although not immediately obvious, a woman’s motivation to get antenatal care reflects her participation in decision-making and her overall say in pregnancy related health behaviors [35]. The IHDS-II question on antenatal care was straightforward: it asked the woman if she had received an antenatal check-up during her last birth (yes/no). It is important to note that this question on antenatal care masks individual, family and community level motivation (or constraints) associated with women’s antenatal health. However, due to unavailability of a comparable theoretical construct, the antenatal care variable has been retained as the primary independent variable for this study. This caveat needs to be acknowledged while interpreting the association between a self-compassion based empowerment measure and utilization of antenatal care.

#### 2.3.3 Covariates

The control variables used in the analysis included woman’s socio-demographic characteristics including her age, education (defined as a 5-category variable ranging from no education to someone who has a graduate degree), marital status (a dummy taking the value of 1 if currently married and 0 if divorced, widowed or separated from spouse). Household characteristics included her relation to the household head (a dummy variable that took the value of 1 if she is the wife of the head, and 0 otherwise) and a household structure variable that used the number of married females as a proxy for joint (or extended) versus a nuclear household. In the model, a “joint” dummy variable was included which is 1 if total number of married females in the household is greater than 1 and 0 otherwise. Conceptualizing the household structure variable through the presence (or lack thereof) of other married females in not new in demographic scholarship [36,37]; in fact, this conceptualization is also significant from the perspective of women’s empowerment since same research has demonstrated that presence of other (older) females is often associated with decreased freedoms in terms of paid work, mobility and overall decision-making since younger women are expected to be compliant to family approvals and surveillance. Finally, a household wealth had been captured using five quintiles based on the household assets variable available in IHDS-II. The asset variable was a scale that sums 33 dichotomous items measuring household possession and household quality (including presence of a vehicle, television, refrigerator, pressure cooker, telephone, etc. to the presence of indoor piped water, *pucca* (solid or concrete) roof, *pucca* wall, etc.). The constructed quintile variable in this paper took the value of 1 through 5, with 1 being the “poorest” and 5 being the “richest” of the households in the sample. At the community level, social group affiliation (caste categories), place of residence (rural versus urban) and religion have been included in the empirical model. As such, India has a stratified society and social distances in terms of residential segregation, language, identity formation and occupations are still organized around caste (upper caste Brahmins versus lower castes including Scheduled Tribes, STs; Scheduled Castes, SCs and Other Backward Castes or OBCs) and communal lines [38,39]. Specifically, caste information in IHDS-II was captured in 6 self-reported categories of Scheduled Tribes (STs), Scheduled Caste (SCs, *dalits*), Other Backward Classes (OBCs), Brahmins-who are traditionally at the top of the caste hierarchy, Forward castes (except Brahmin) and a residual “Other” category. For the purpose of this analysis, caste variable was recoded to include the Brahmin, Forward and the residual “other” category and relabeled as the “general” category (a term typically used to differentiate these socially privileged caste categories from their “lower” counterparts with certain employment and education advantages associated with the non-general caste categories) while preserving the remaining three categories (ST, SC and OBC). The respective percentages of the caste categories in the final model are General (30 percent), OBC (41 percent), SC (21.33 percent) and ST (8.30 percent). IHDS-II provides religion information of the household through seven commonly reported categories (Hindu, Muslim, Christian, Sikh, Buddhist, Jain, Tribal), plus a residual “other” category and “none”. The religion variable has been recoded into 5 major categories of Hindu (82 percent), Muslim (12 percent), Christian (2.34 percent), Sikh (2.39 percent) and an “other” category (1.40 percent) that included the remaining 5 religious categories that were numerically small. In the OLS model dummies were used with Hindu being the reference category for religion, the “general” category being the reference group for caste and urban for the place of residence variable. Finally, all state level unobserved social and cultural differentials were controlled for by including 34 state dummies.

### 2.4 Analytical strategy

In the next stage, as part of the multivariable model, an Ordinary Least Squares (OLS) was performed with empowerment as the dependent variable and antenatal care (for last birth) as primary independent variable. OLS estimation is a more appropriate statistical technique than an ordered Probit model (typically used for ordinal outcomes). The regression equation (when the empowerment measure is regressed on antenatal care) can be represented as follows:

Empowerment=β1antenatalcare+β(PC)+β(HC)+β(SC)+ε

where, vector *PC* denotes personal characteristics (including respondent’s education, age, marital status, age at marriage), vector *HC* denotes household characteristics (including caste, religion, relationship to household head, number of other married women in the household, place of residence, household asset quintiles) and *SC* denotes state level characteristics (includes 33 state dummies).

## 3. Results

### 3.1 Summary statistics through means comparisons

Table 4 offers a closer look at the empowerment measure across selected control variables thereby offering a descriptive portrait of the data. Overall, the means comparisons confirmed pervious scholarship on the gains that a woman typically makes in empowerment when located in an urban area or belonging to an Hindu household or being older when compared to a woman residing in a rural area, belonging to a Muslim household or being at the bottom of the age hierarchy within the household (see [26]). Again, it is conceivable that women who report higher values in self-compassionate empowerment are also the ones who report higher overall wellbeing (i.e. self-reported overall health). What is noteworthy, however, is the somewhat incongruent association between caste categories and wealth. Specifically, the self-compassionate empowerment measure was only marginally higher for the “general” category when compared to those belonging to the OBC households, but these marginal gains are eroded when compared to those in the SC & ST households. Similar patterns were evident in the wealth quintiles, where household economic status seemed to be somewhat unrelated to a woman’s empowerment. In fact, in Table 4, the women in the richest households reported having lower values of self-compassionate empowerment when compared to less-wealthier households in the middle and the 4^th^ quintiles. Although these associations were not controlled for other sociodemographic and household characteristics of the women, but what is seemed to suggest is that social and economic privileges (through higher caste affiliation or higher household economic status) do not follow the conventional pathways when it comes to understanding women’s empowerment. In fact, Kabeer ([6]: cited in [26]) pointedly noted this dilemma by arguing that women’s agency is different from other welfare related issues since it lies at the “nebulous territory of power and social injustice” (p. 435).

**Table 4:** The self-compassion empowerment (mean) values across selected control variables.

*Place of residence*	Mean Empowerment score (S.D)
Rural	2.56 (1.82)
Urban	2.71 (1.83)
*Caste Categories*	
ST	2.57 (1.76)
SC	2.70(1.86)
OBC	2.58(1.88)
General	2.62(1.69)
*Religion*	
Hindu	2.63 (1.83)
Muslim	2.42(1.72)
Christian	3.21(2.07)
Sikh	2.35(1.56)
Others	2.93(1.90)
*Age Categories*	
15-19years	1.29 (1.25)
20-24years	1.73(1.37)
25-29years	2.21(1.54)
30-40years	2.71(1.72)
41&above	3.06(1.99)
*Asset Groups(Quintiles)*	
Poorest	2.46 (1.79)
2nd Quintile	2.63(1.88)
Middle	2.75(1.90)
4th Quintile	2.69(1.85)
Richest	2.55(1.66)
*self-reported overall health*	
Good	2.65 (1.80)
Okay	2.67 (1.86)
Poor	2.53(1.96)

Source: IHDS (2011–12)

### 3.2 Results from OLS estimates

Table 5 presents results for the OLS estimates.

**Table 5:** OLS estimates (*unstandardized β coefficients*), IHDS-II.

Predictor variables	Self-compassionate empowerment	95% conf interval
Antenatal Care (yes = 1)	0.219***	(.135;.302)
*Personal characteristics*		
Primary *(no education)*	0.329***	(.212; .446)
High school	0.311***	(.241;.381)
some college	0.463**	(.349;.576)
graduate	0.467***	(.334;.599)
Age15-19 *(age25-29)*	-0.576*	(-.778;-.373)
Age20-24	-0.332**	(-.402;-.263)
Age30-40	0.304**	(.242;.366)
Age41&above	0.521***	(.362; .678)
Married *(divorced/separated/widowed)*	-0.342*	(-2.12; -1.64)
age at marriage	-0.015	(-.014; .003)
*Household characteristics*		
Wife of household head (= 1, if yes, 0 otherwise)	0.017	(-.066; .081)
Lives in a Joint family	-0.359**	(-.433; -.284)
poorest *(q3 = middle)*	-0.081*	(-.165; .002)
q2	-0.013*	(-.099;.072)
q4	0.014	(-.071; .099)
Richest	-0.019**	(-.291;-.091)
*Community characteristics*		
Muslim *(Hindu)*	-0.041	(-.117; .036)
Christian	0.332**	(.144; .520)
Sikh	-0.244**	(-.423; -.064)
Other Religion	0.217*	(-.023; .458)
OBC *(General)*	-0.045	(-.111; .019)
ST	0.123*	(.014;.228)
SC	0.098*	(.015; .172)
urban *(rural)*	0.065	(-.058; .071)
Constant	3.87***	
Observations	36,427	
Adj R-squared	0.079	

IHDS (2011–12).

*p< .05

**p< .01

***p < .001; Reference categories for each variable appear in italics. State controls are not reported here.

As hypothesized earlier, antenatal care was positive and significant suggesting a possible pathway through which empowerment as self-compassion improves a woman’s self-efficacy in terms of pregnancy care (b = 0.219; C.I = .135, .302; p = 0.001). This positive and statistically significant result remained even after controlling for household economic status suggesting that antenatal care was not just a function of household resource but had an important role in increasing the woman’s capacity to be self-compassionate. Personal characteristics of the woman such as her educational attainment and age were in the expected direction. For example, higher levels of education (e.g. for a graduate degree, b = 0.467; C.I = .334, .599; p = .001) was associated with relatively higher attention to her own self while these gains were reversed when she is a younger woman (e.g. for age group 20–24 years, b = -.332; C.I = -.402, -.263; p = .01) with limited power in decision-making for herself and her family. Although not significant, the negative association between age at marriage and empowerment could be due to a non-linear, quadratic relationship between the two [26] where a woman’s self-interested empowerment increases with her age at first marriage but may fall or become redundant after attaining a maxima at a certain age. Again, being “married” has a statistically significant negative association with self-compassionate empowerment (b = -.342; C.I = -2.12, -1.64; p = .05).

In terms of community characteristics, as seen previously in the means comparisons results, the findings here are not very straightforward underscoring the complex pathways through which woman’s attention to herself is lived and experienced. For example, while being in a Muslim household negatively affected her empowerment (when compared to those belonging to the Hindu households), the relationship was not statistically significant (b = -.041; C.I = -.117, .036). Again, while belonging to the SC (b = 0,098; C.I = .015, .172; p = .05) and ST (b = .123,; C.I = .014, .228; p = .05) caste categories were positively associated with her empowerment, belonging to OBC was not. The urban advantage, as observed in a majority of demographic scholarship on India in terms of women’s empowerment and health outcomes was not found in this model. Meanwhile, when compared in relation to the middle quintile (Q3), women’s empowerment remained either unaffected or negatively associated with economic status of the household.

Finally, the positive association between a self-compassionate based empowerment and antenatal care resonated well with the overall outcomes of women as observed in state-level demographic analysis in India (see [40] for a careful examination of social inequalities spatially). For example, when compared to Jammu & Kashmir, the southern states of Kerala, Karnataka and Andhra Pradesh as well as the northeastern states of Nagaland, Manipur, Mizoram (with the exception of Arunachal Pradesh) showed a positive association between self-compassion and antenatal care, whereas states of Jharkhand, Orissa, Chattisgarh, Madhya Pradesh and Gujarat (demonstrated evidence of poorer demographic outcomes in terms of maternal and child health) recorded a negative association between the two. While these results were largely consistent with the socio-demographic realities of these states, the coefficients were statistically non-significant in the model (and hence not reported in Table 5).

## 4. Discussion

This study investigated the association between women’s empowerment conceptualized through a framework of self-compassion and its antecedents. This study had two goals: First, to bring empowerment, understood through the lens of self-compassion, under empirical scrutiny and investigate its association with antenatal health and attendant covariates; second, paying attention to the psychometric quality of this measure. Given the conceptual ambiguities embedded in the multidimensional nature of women’s empowerment, a confirmatory factor analysis (CFA) had been conducted to ensure that the identified theoretical constructs fit the overall data well. This process was followed by reliability and validity exercises. Ultimately, the empowerment measure derived after the CFA was used in the OLS estimation.

Few results deserve attention. In addition to a positive association between empowerment and antenatal care, there was an age and education gradient in empowerment. Put simply, individual level influences on empowerment can be dissipated by household level influences. That is, while education might enhance gains in empowerment but those effects might be muted if the woman resides in a joint household as opposed to a nuclear one. The negative and significant “married” dummy was surprising since having a husband has been shown to be associated with positive health outcomes through increased say in decision making for women. This unexpected result might be explained in relation to a negative and a significant “joint” household variable, which might point to a relatively more important role played by the (older) female than the spouse when it came to the woman’s capacity to negotiate household decisions in her own favor (mobility, social network, etc.). In fact, The results of the OLS estimates remain unchanged when the joint variable is recoded to control for the age of the other married females in the household (results not reported here). In particular, the recoded joint variable dummy variable is 1 if total number of married females in the household is greater than 1 and is of age greater than 40 years and 0 otherwise. The choice of 40 years was taken since it is not uncommon in India to become a mother-in-law in late forties given high rates of early marriage and pregnancy in India. This explanation can be supported by Desai & Banerji’s [41] study on the impact of husbands’ migration on left-behind wives in India where the authors note that household structure (extended versus nuclear) in the key mediating factor that affects women’s autonomy. In particular, their study shows that women in migrant households (one where the husband has migrated) have higher predicted average autonomy scores where no older woman is present as opposed to those in extended households with an older woman. Again, the same study reports higher levels of autonomy (reflected in predicted average scores) for divorced/widowed women than those who are currently married. This relatively higher autonomy for divorced and widowed women, the authors explain, could be a result of a greater likelihood of freedom to make decisions concerning them or their children in the absence of a male head. In summary, these results might explain why women’s self-compassionate empowerment did not necessarily have a positive association with her “married” status and by that extension it had no statistically significant bearing on her relationship (as a wife) to the household head.

Again, consistent with other studies [26] results suggested that social group affiliation on women’s empowerment was mixed and at best ambiguous. For example, the higher social caste advantage did not play out in a straightforward way as it did with other demographic outcomes (e.g. child nutrition or maternal mortality). One explanation could be that the empowerment effect is moderated by the household structure variable. That is, studies show that women from upper social castes are more likely to reside in joint families (known to restrict women’s freedoms) than lower social castes (OBCs, SC, STs; see [37]) which might explain the not-so-obvious direction of these relationships. Additionally, one can argue that demands of family honor and social prestige are more firmly lodged in the creation of an upper caste womanhood [42] that may ultimately constrict women’s household bargaining power, mobility and sociality. Again, the unexpected finding of a negative association of urban residence with a self-compassionate based empowerment could be explained by restrictions on mobility (for work or leisure) experienced by urban women in contemporary India. Although a rural-urban comparative analysis of women’s freedom of movement is absent from the sociological scholarship, feminist scholars have shown how women’s mobility in urban India remains circumscribed within the normative boundaries of class, community, gender and sexuality [43,44]. Hence, it is perhaps no surprise that the urban residence was not associated with gains in a self-compassionate based empowerment measure. Similar observations could be made with respect to religion and its influence on women’s empowerment. What seems surprising, but commonly observed in the development scholarship is that household wealth does not necessarily trickle down to women [6,45]. In the OLS estimation, affluence (measured through asset quintiles) showed no discernable benefit for women’s attention to her own self. In fact, when compared with the middle class households, both the poorest and the richest made no gains in empowerment. Significantly, this ambiguous relationship between wealth and empowerment revealed the complex intersections of social norms and gender and offers evidence to the contradictions tensions of how womanhood in India is lived and experienced.

Overall, this study offered a useful starting point to look at empowerment through a relatively unexplored area of social psychology. For a more nuanced analysis, this would have involved looking at a woman’s access to her own leisure, dietary choices, self-interested life goals and aspirations which when taken together would improve her overall quality of life through self-efficacy and self-awareness. Future data collection efforts in India could incorporate time-use frameworks, which could provide a detailed distribution of time that women might spend on self-interested goals as opposed to delivering domestic duties.

### 4.1 Limitations

A few limitations of the study might be noted. As mentioned earlier, the notion of self-compassion has conceptual roots in social-psychological research and is often associated with psychological indices (e.g. less anxiety, social-connectedness and life satisfaction). Unfortunately, data from India do not allow for any measurement of psychological wellbeing, feelings or affect, which would been most appropriate to investigate the empirical utility of a self-compassion based empowerment measure. While being completely aware of this limitation, this study utilized some of self-compassion’s well-established *indirect* links to assertiveness, autonomy and positive physical health behaviors [15,16,28,46]. The IHDS data, with its unique focus on intra-household gender dynamics and reproductive health, was found to be best possible alternative in empirically harnessing these indirect routes. Hence, although the CFA allowed testing each dimension of self-compassionate empowerment (as opposed to summed scales), this caveat needs to kept in mind while interpreting the results. Finally, a note on the foundational assumption in the conceptual framing of empowerment as self-compassion is warranted. Drawing from their anthropological fieldwork in Punjab (Pakistan), authors Mumtaz & Salway [47] in their penetrative critique of the “autonomy paradigm” point out to a distinct lack of cultural specificity, inadequate attention to masculinities and a disregard for gender relations in the conceptualization and measurement of women’s autonomy/empowerment in the development discourse. In particular, they emphasize the cultural preeminence of “community rather than individuality as a social ethic”. The implication of this understanding, the authors argue, lies in a recognition that “western” and South Asian personhood are different such that the notion of “self” may devalue the relationships of interdependence (e.g. loyalty, caring and responsibility)-known to be culturally important in South Asia. Although the theoretical relevance of anthropological work cannot be directly translated into empirical questioning, this conceptual caveat should be kept in mind while recasting the empowerment debate in terms of *self*.

## 5. Conclusion

Overall, findings from the OLS models corroborate demographic and sociological literature on women’s position within the household in India. In particular, this study demonstrated that while there was a statistically significant positive association between a self-compassion based empowerment and reproductive health (antenatal care), women’s position remained largely circumscribed within the contradictory pulls of family structure and social class with no evident gains from being married, belonging to an upper caste, or residing in an affluent household in an urban area.

Finally, this study also highlighted the researcher’s dilemma in being caught between asking a relatively old question through a newer conceptual framing and adopting a statistically robust method to offer a valid and stable measure of empowerment (The recent debate around the methodological dissatisfaction around a cross-culturally stable index, SWPER (Survey-based women’s empowerment) claimed to improve Sustainable Development Goal 5 (gender equality) exemplifies this dilemma (see Yount, et al. [48] critique of the measure and later, the authors reply (Barros, et al. [49] in Lancet.) For example, due to appropriate data availability and the reliability analysis as part of the CFA, this study could not employ some of the more theoretically relevant dimensions of empowerment (e.g. decision regarding spousal choice, contraceptive preference, name of household ownership documents).

These caveats notwithstanding, this study offered a critique of the standard survey-based gender questions in the following ways: First, results from this study showed that the experience of empowerment could coexist with other constraints; second, this study complicated the notion of domestic responsibility by attempting to disentangle the self from its instrumental components; and finally, as noted earlier, this study was a call to develop instruments to understand and measure the intimate link between the less visible markers of empowerment such as self-compassion, negotiation, conditions of individual choice or as Basu & Koolwal [9] astutely put it- “unproductive freedoms”.
